# A comparative analysis of pollinator type and pollen ornamentation in the Araceae and the Arecaceae, two unrelated families of the monocots

**DOI:** 10.1186/1756-0500-2-145

**Published:** 2009-07-22

**Authors:** Julie Sannier, William J Baker, Marie-Charlotte Anstett, Sophie Nadot

**Affiliations:** 1 Univ Paris-Sud, Laboratoire Ecologie, Systématique et Evolution, UMR 9079, Orsay, F-91405, France; 2 Royal Botanic Gardens, Kew, Richmond, Surrey, TW9 3AB, UK; 3 Centre d'Écologie Fonctionnelle et Évolutive, Centre National de la Recherche Scientifique, 1919 Route de Mende, 34293 Montpellier Cedex 05, France

## Abstract

**Background:**

The high diversity of ornamentation type in pollen grains of angiosperms has often been suggested to be linked to diversity in pollination systems. It is commonly stated that smooth pollen grains are associated with wind or water pollination while sculptured pollen grains are associated with biotic pollination. We tested the statistical significance of an association between pollen ornamentation and pollination system in two families of the monocotyledons, the Araceae and the Arecaceae, taking into account the phylogenetic framework.

**Findings:**

Character optimization was carried out with the Maximum Parsimony method and two different methods of comparative analysis were used: the Concentrated-Change test and the Discrete method. The ancestral ornamentation in Araceae is foveolate/reticulate. It is probably the same in Arecaceae. The ancestral flowers of Araceae were pollinated by beetles while ancestral pollination in Arecaceae is equivocal. A correlation between ornamentation type and pollination was highlighted in Araceae although the results slightly differ depending on the method and the options chosen for performing the analyses. No correlation was found in palms.

**Conclusion:**

In this study, we show that the relationships between the ornamentation type and the pollination system depend on the family and hence vary among taxonomic groups. We also show that the method chosen may strongly influence the results.

## Findings

The exine wall of the pollen grains of flowering plants displays patterns of ornamentation (the external aspect of pollen grains, also called sculpturing) that are highly diversified. The reasons accounting for such variation in the ornamentation of pollen grains in flowering plants still remain unclear. Among the different types of relationship implying pollen ornamentation that have been suggested, the existence of a link between exine sculpturing and pollinator type has often been proposed and was even evidenced in certain situations (see additional file 1). It is often considered that smooth pollen grains are associated with abiotic pollination (wind or water) while echinulate or reticulate pollen grains are associated with biotic pollination, particularly entomophily [1,2]. These results show that the adaptiveness of this character still remains largely debated.

The study presented here aims to test the hypothesis suggested by Grayum [3] concerning a relationship between pollen ornamentation and pollinator type in the Araceae, using Phylogenetic Comparative Methods. He established a correlation between (a) psilate and verrucate pollen and pollination by beetles and (b) echinulate pollen and pollination by flies. We think that the flaw of this study is inherent to the fact that correlations were established without statistical analysis and without taking into account the phylogenetic background of the family, making it impossible to know whether the correlations observed between the pollen and pollinator types result from adaptation or from common ancestry.

The processes underlying a relationship between two characters remain generally extremely difficult to determine [4,5]. A correlation may be the result of adaptation, but also of developmental constraints. It may also be simply the result of phylogenetic inertia *i.e*., that related species resemble each other more than they resemble species drawn at random [6]. Various mathematical approaches, called Phylogenetic Comparative Methods or PCM [4,7], have been proposed over the last twenty years [8-10] and take into account the phylogenetic background of the organisms studied.

Here we re-examine the correlation between pollen sculpturing and pollinator type proposed by Grayum [3], in light of the phylogenetic framework available for the Araceae family [11] using two PCM applied to discrete characters. In the conclusion of his paper, Grayum suggested to investigate other groups of monocotyledons, palms in particular. In this family a large amount of pollen data has been recorded but rarely studied from an evolutionary point of view, except for the number of apertures [12]. Moreover data on pollinators are available and a detailed and well resolved phylogeny including almost all of the genera [13] now exists. Consequently we also examine the correlation between pollen and pollinator types in the palm family (Arecaceae).

**Methods (for details, see additional file **2**)**

Character optimization was carried out with the Maximum Parsimony method implemented in the Mesquite software [14].

Two PCMs were used: the Concentrated Changes Test or CCT [9] and Discrete [10].

## Results and discussion

### Character evolution in the Araceae

**Figure 1:**
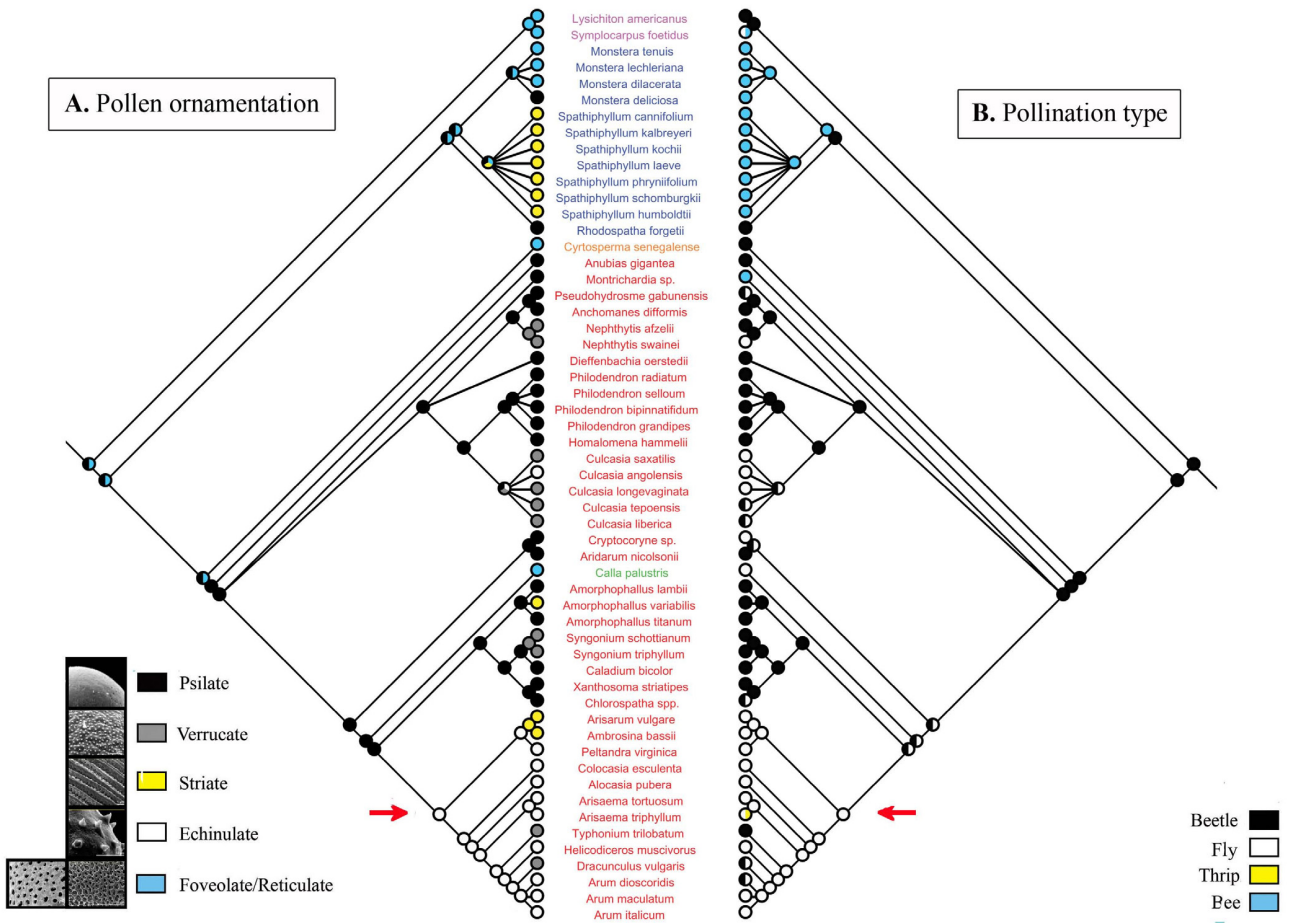
**Optimization of the evolution of ornamentation and of pollination in the Araceae**. Composite phylogenetic tree of the family where each mirror tree presents the optimization of one character. A. Optimization of ornamentation type (five character states: Psilate, Verrucate, Striate, Echinulate and Foveolate/Reticulate). Pictures are given as illustratiion for each of these types, they do not correspond to a particular species of the family. They were obtained from . B. Optimization of pollination type (four character states: Beetle, Fly, Thrip and Bee pollination). Species names are coloured according to the subfamilies (Orontioideae in pink, Monsteroideae in blue, Lasioideae in orange, Calloideae in green and Aroideae in red). The last common ancestor of the group Areae+Arisaemateae is indicated by a red arrow.

To our knowledge, there is little data in the literature concerning the evolution of ornamentation of pollen grains in monocots [15]. Concerning the angiosperms, a recent study showed that the ancestral exine structure had a continuous or microperforate surface [16]. However, foveolate-reticulate tectum would have arisen soon after [16]. The work of Grayum [17] that is re-examined here, provides hypotheses about the ancestral and derived states of pollen wall sculpturing within the Araceae (monocots). His proposition that the most primitive aroid pollen had foveolate to reticulate exine is not in contradiction with our phylogenetic analysis of the character. Indeed, our results suggested that the hypothetical aroid pollen was either 'Foveolate/Reticulate' or 'Psilate' for pollen ornamentation (Figure 1A). The equivocal ancestral state is probably due to the polytomies, coded as soft (uncertainty in resolution), that are present in the tree. From this equivocal type, different types of sculpturing evolved [17]. However, no type of ornamentation is restricted to one clade and no particular trend in the evolution of the character emerged clearly from our analysis. It can be noted that several ornamentations originated several times independently and most of all, from different character states. This indicates that some transitions may occur indifferently among the different states.

Concerning the pollination type, optimization of character evolution suggested that the last common ancestor of the family was pollinated by 'Beetle' (Figure 1B), according to outgroup comparison with other Alismatales [17]. From this condition, the other types of pollination each evolved several times within the family. In particular, fly pollination is clearly derived from beetle pollination in Aroideae, where it evolved in several unrelated genera and is synapomorphic for the *Arisaemateae*+*Areae *clade (indicated by a red arrow in Figure 1).

**Figure 2:**
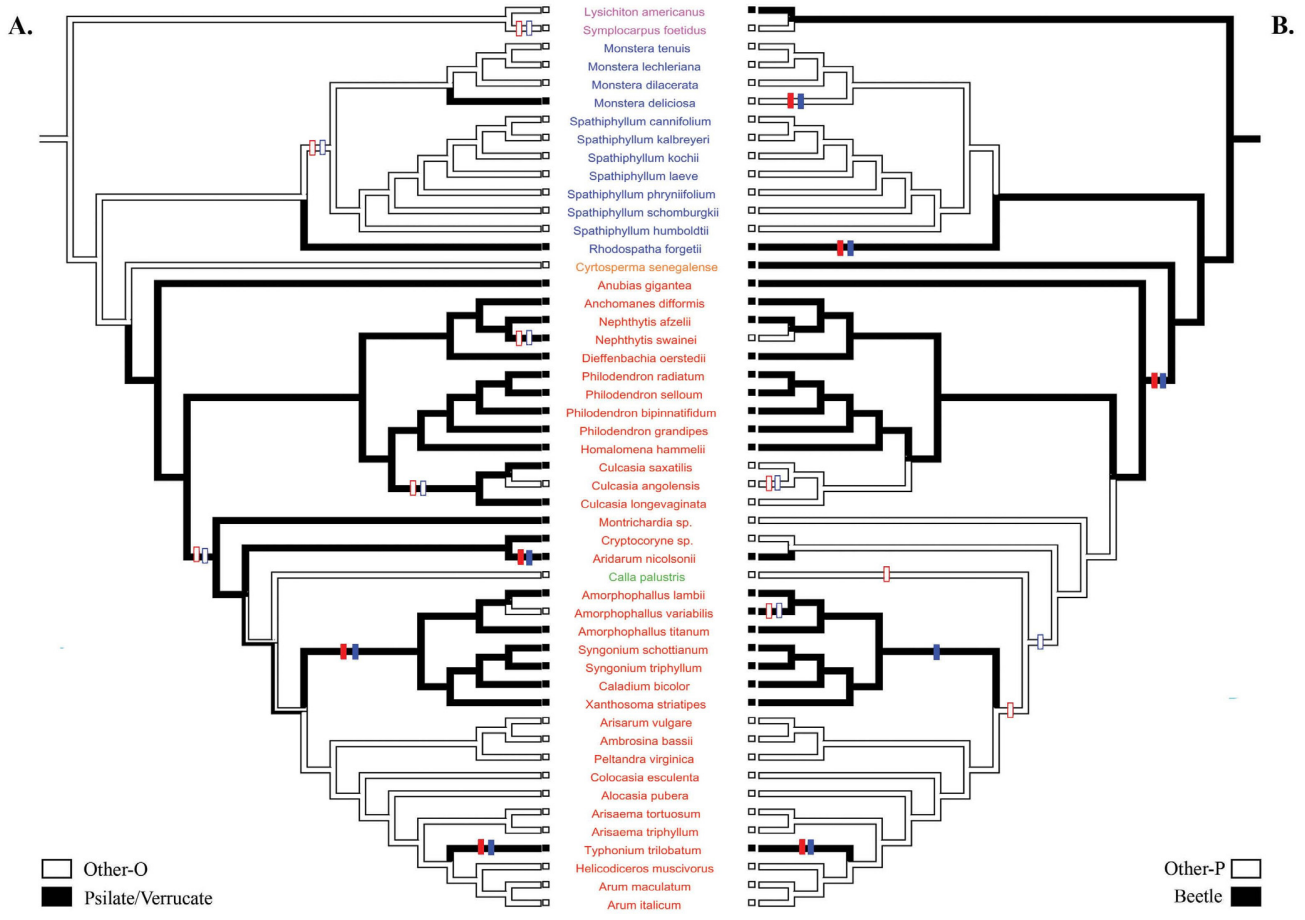
**Evolution of ornamentation and pollination in Araceae with polymorphic species removed**. A. Optimization of ornamentation type coded as 'Other-O' (white) and 'Psilate/Verrucate' (black). B. Optimization of pollination type coded as 'Other-P' (white) and 'Beetle' (black). The bicoloured branches indicate an equivocal inference of the ancestral character state. The transitions towards 'Beetle' pollination and 'Psilate/Verrucate' ornamentation are indicated by full crossbars and the reversals towards 'Other-P' pollination and 'Other-O' ornamentation are indicated by open crossbars (red and blue crossbars correspond respectively to ACCTRAN and DELTRAN optimizations). Species names are coloured according to the subfamilies (Orontioideae in pink, Monsteroideae in blue, Lasioideae in orange, Calloideae in green and Aroideae in red).

**Figure 3:**
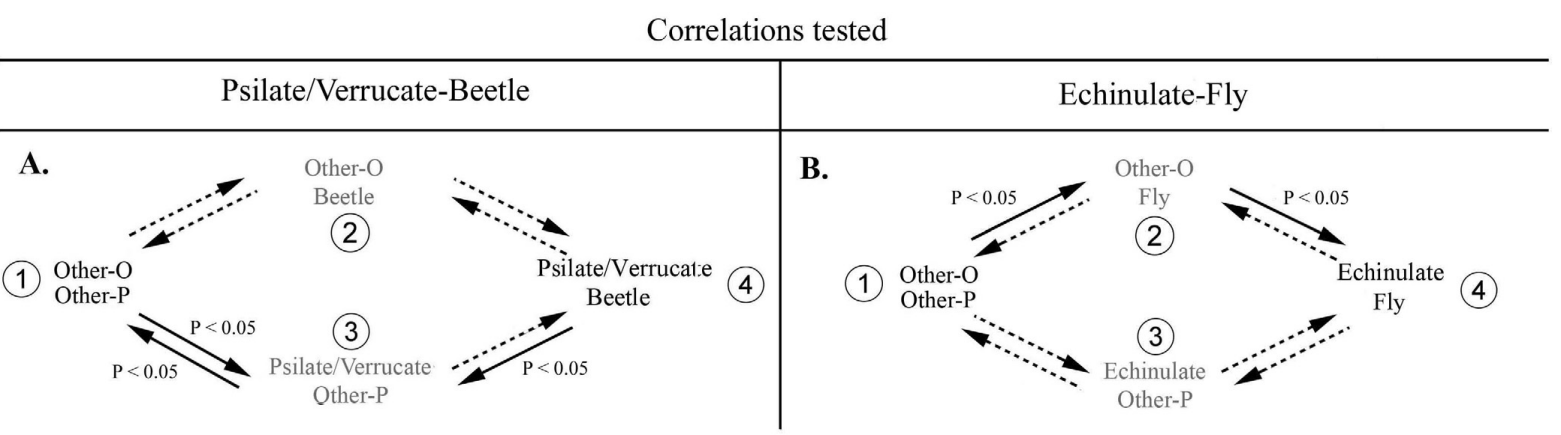
**Flow diagrams of correlated evolution between ornamentation and pollination in Araceae and Arecaceae**. Flow diagrams of correlated evolution between 'Psilate/Verrucate' ornamentation and 'Beetle' pollination (A-B) and between 'Echinulate' ornamentation and 'Fly' pollination (C-D) in the Araceae. Solid arrows indicate significant transitions; dotted arrows indicate non-significant transitions. The larger the arrow, the greater the level of significance. The numbers correspond to the different situations. The situations in gray correspond to transitional intermediate states. A-C: polymorphic species duplicated. B-D: polymorphic species removed.

After the transformation of the coding from multistate to binary characters (Figure 2 and additional files 3, 4 and 5), we sought to test a correlation between pollen ornamentation and pollinators using the concentrated-change test [9] and the discrete method [10]. When polymorphic species were removed, both methods found a correlation between the ornamentation and the pollination (Tables 1 and 2, additional files 6 and 7). The Echinulate type was found as contingent upon Fly pollination (changes towards Echinulate pollen happened more often in Fly pollinated taxa; Table 1) with the CCT only. However, with the Discrete method, the flow diagram showed that transitions towards Fly pollination were probably followed by transitions towards Echinulate ornamentation (Figure 3).

When polymorphic species were duplicated, a correlation was found between ornamentation and pollination only using the CCT. With the coding 'Echinulate' ornamentation and 'Fly' pollination, the correlation was significant when the ornamentation was considered as dependent of the pollinator (Table 2, additional files 6 and 7).

**Table 1:** Comparative analyses conducted with the Concentrated-Changes Test [9] in Araceae.

		P-value
Coding 1	P/V → B	P < 0.05
	
	B → P/V	P < 0.05

Coding 2	E → F	NS
	
	F → E	P < 0.05

P-values obtained through a Fisher's exact test performed on the distribution of events with the coding 1 ('Psilate/Verrucate' (P/V) vs. 'Other Ornamentation' and 'Beetle' (B) vs 'Other Pollination') and the coding 2 ('Echinulate' (E) vs. 'Other Ornamentation' and 'Fly' (F) vs 'Other Pollination'). The results are given when the character 'pollination type' depends on the 'ornamentation type' (P/V → B, E → F) and conversely (B → P/V, F → E) and only with ACCTRAN optimization (complete and detailed results in additional files 6 and 7). Polymorphic species were removed.

**Table 2:** Comparative analyses performed using Discrete.

Family	Polymorphic species	Type of test	|LR|	df	P
Araceae	Removed	• Omnibus test (P/V correlated with B)	12.80	4	< 0.05
		
		○ Contingent change test:			
		
		P/V → B	2.90	1	NS
		
		B → P/V	0.77	1	NS
		
		◊ Temporal order test	1.78	1	NS
		
		• Omnibus test (E correlated with F)	15.34	4	< 0.01
		
		○ Contingent change test:			
		
		E → F	0	1	NS
		
		F → E	0.28	1	NS
		
		◊ Temporal order test	0.33	1	NS
	
	Duplicated	• Omnibus test (P/V correlated with B)	0.86	4	NS
		
		• Omnibus test (E correlated with F)	0.29	4	NS

Arecaceae	Duplicated	• Omnibus test (P/V correlated with B)	2.61	4	NS
		
		• Omnibus test (E correlated with F)	0.3	4	NS

List of the different tests performed in Araceae and Arecaceae depending on the phylogeny used (polymorphic species removed or duplicated). LR = likelihood ratio. P/V: Psilate/Verrucate ornamentation; E: Echinulate ornamentation; B: Beetle-pollination; F: Fly-pollination.

Note: arrows in contingency tests mean that the changes in one variable are contingent on one state in the other variable; for example, P/V → B means that changes from 'Other' to 'Beetle' pollination are more likely to occur when the ornamentation is 'Psilate/Verrucate'. Omnibus tests analyze the correlation between two binary discrete characters. Contingent tests analyze whether a state of one trait favours the evolution in the second trait. Temporal order tests analyze whether the change in one trait occurred significantly before the change in the other trait. The results are summarized in the flow diagrams (Figure 5).

These results indicate that the impact of adding/removing information on the detection of a correlation varies according to the method used (CCT or Discrete). The interpretation of the results may then be strongly influenced by the method chosen, as already shown [18]. In our case, duplicating polymorphic species leads to increase the number of opposing associations (associations for which we do not seek correlation). However, treat polymorphic species as pairs of species (duplicate) with contrasting character states [19] is the most conservative option and avoids loss of information. In conclusion, it is important to be aware of this problematic when a choice has to be made.

### Character evolution in the Arecaceae

**Figure 4:**
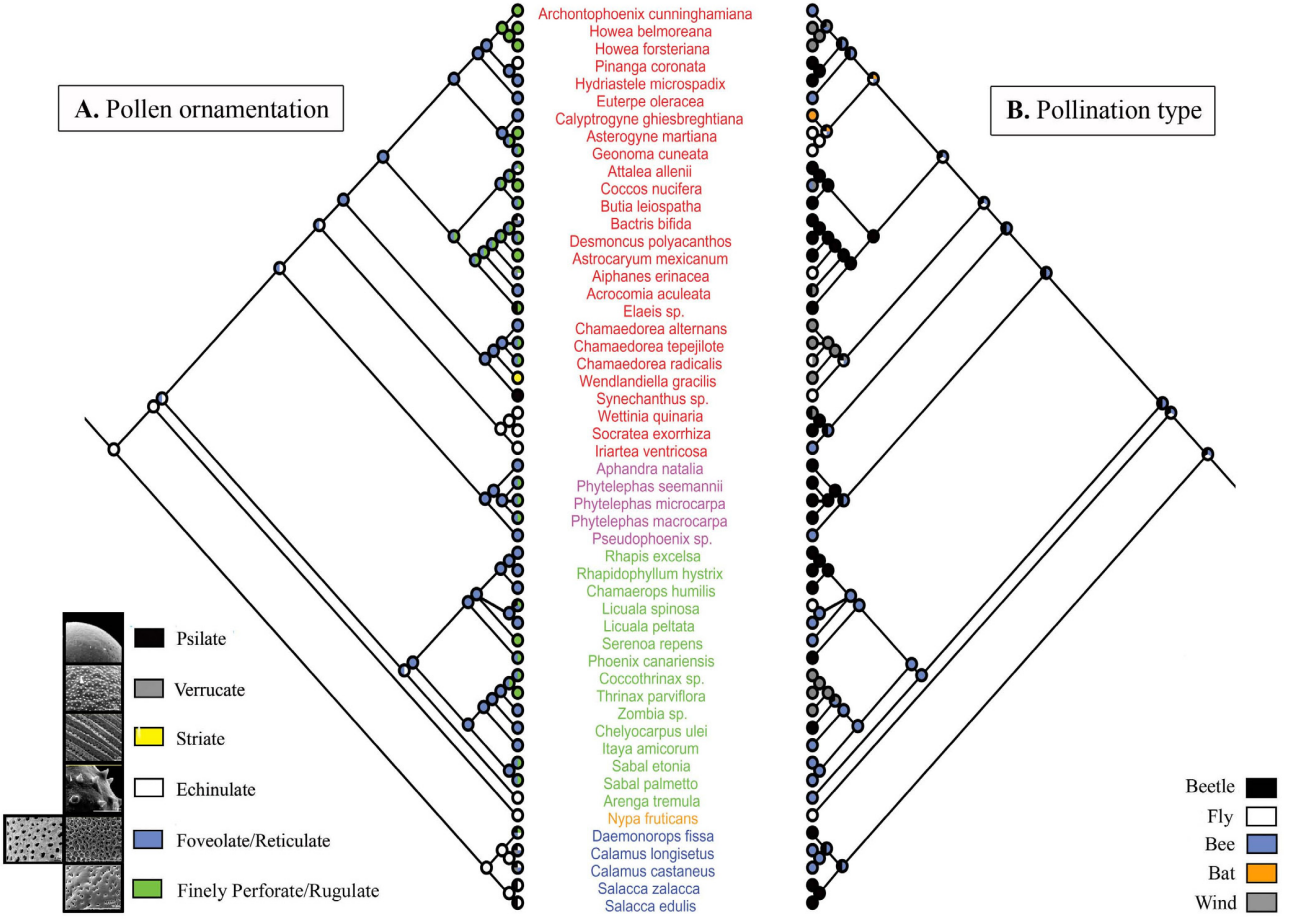
**Optimization of the evolution of ornamentation and pollination in Arecaceae**. Supertree of Arecaceae where each mirror tree presents the optimization of one character. A. Optimization of ornamentation type (five character states: Psilate, Verrucate, Striate, Echinulate and Foveolate/Reticulate). Pictures are given as illustration for each of these types, they do not correspond to a particular species of the family. They were obtained from . B. Optimization of pollination type (five character states: Beetle, Fly, Bee, Bat and Wind pollination). Species names are coloured according to the subfamilies (Calamoideae in blue, Nypoideae in orange, Coryphoideae in green, Ceroxyloideae in pink and Arecoideae in red).

In spite of the important literature on pollen ornamentation available for the Arecaceae [20], no steady hypotheses have been proposed about the ancestral ornamentation, apart from a suggestion that psilate exine could be the primitive condition [21]. The present study is the first that makes hypotheses about the ancestral features of pollen grain in palms using a phylogenetic approach. In our analysis, the ancestral character state for the family was inferred as 'Echinulate' (Figure 4A). However, the reconstruction of the ornamentation character on a phylogeny of the family including all of the genera inferred a 'Foveolate/Reticulate' ancestral character state (personal information). This conflict is explained by the presence of only polymorphic genera in Calamoideae in our study.

**Figure 5:**
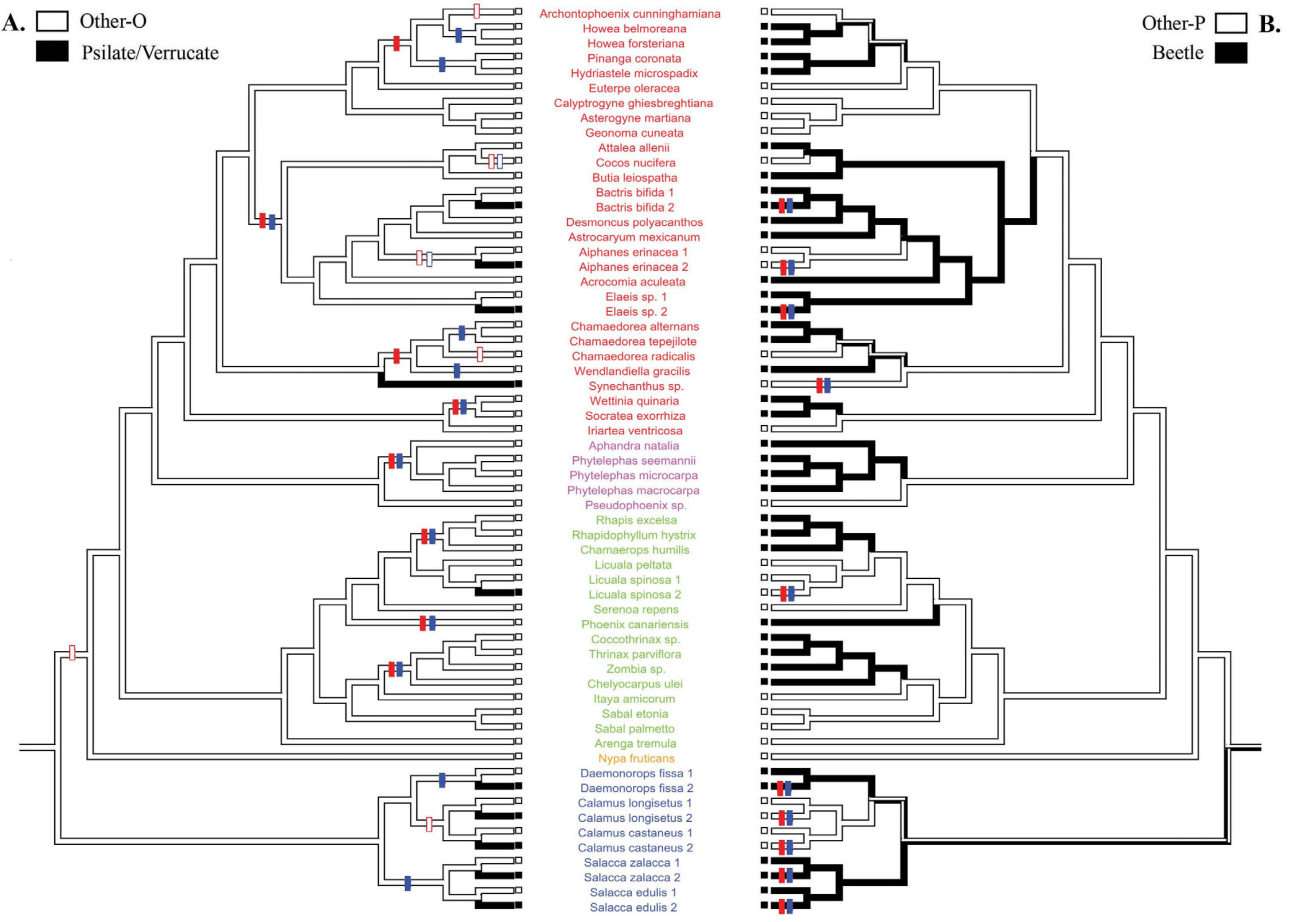
**Evolution of ornamentation and pollination in Arecaceae with polymorphic species duplicated**. A. Optimization of ornamentation type coded as 'Other-O' (white) and 'Psilate/Verrucate' (black). B. Optimization of pollination type coded as 'Other-P' (white) and 'Beetle' (black). The bicoloured branches indicate an equivocal inference of the ancestral character state. The transitions towards 'Beetle' pollination and 'Psilate/Verrucate' ornamentation are indicated by full crossbars and the reversals towards 'Other-P' pollination and 'Other-O' ornamentation are indicated by open crossbars (red and blue crossbars correspond respectively to the ACCTRAN and DELTRAN optimizations). Species names are coloured according to the subfamilies (Calamoideae in blue, Nypoideae in orange, Coryphoideae in green, Ceroxyloideae in pink and Arecoideae in red).

The Pollination type appeared as a very variable character and consequently, no clear evolutionary trend emerged from the character optimisation (Figure 4B). The ancestral pollinators either were bees, beetles or flies, and the pollination type for each subfamily (except Nypoideae) was ambiguous. In this family, even with the binary coding (Figure 5, additional file 8), polymorphic species were so numerous that when they were removed, according to one of the option chose, there were not enough data left to perform any test. When polymorphic species were duplicated, as a result of the high variability in characters, the comparative analyses failed to detect a correlation between ornamentation type and pollination type in Arecaceae, whatever the method used (Tables 2 and 3, additional files 9 and 10).

**Table 3:** Comparative analyses conducted with the Concentrated-Changes Test [9] in Arecaceae.

		P-value
Coding 1	P/V → B	NS
	
	B → P/V	NS

Coding 2	E → F	NS
	
	F → E	NS

P-values obtained through a Fisher's exact test performed on the distribution of events with the coding 1 ('Psilate/Verrucate' (P/V) vs. 'Other Ornamentation' and 'Beetle' (B) vs 'Other Pollination') and the coding 2 ('Echinulate' (E) vs. 'Other Ornamentation' and 'Fly' (F) vs 'Other Pollination'). The results are given both when the character 'pollination type' depends on the 'ornamentation type' (P/V → B, E → F) and conversely (B → P/V, F → E) and only with ACCTRAN optmization (complete and detailed results in additional files 8 and 9). Polymorphic species were duplicated.

### Relationships between ornamentation type and pollination

The reason why Angiosperms display such a large diversity of pollen ornamentation remains to date rather unclear. Several studies addressing this question have been produced, often leading to conflicting, or at least different, conclusions. Most studies however examine the hypothesis of a link between pollen ornamentation and the pollination system. The underlying idea is that since pollen grains need a vector (biotic or abiotic) to reach the female parts, the pollen surface may play a role in the efficiency of the interaction either with the pollination agent or with the receptive area of the female organs. A relationship between abiotic pollination (wind and water) and smooth (or nearly) pollen grains has been proposed in many studies [1,22] but concerning biotic pollination, the results are more controversial. [2,3,23,24]. According to previously published studies [3,20,24], our results suggest that the relationship between pollen ornamentation and pollinators may actually depend on the taxon. The association between psilate (=smooth) pollen and beetles seems to be rather specific to the Araceae, since entomogamous species are generally thought to produce pollen grains with a deeply sculptured exine [1,23]. The idea is that the sculptures would enhance the adherence of the pollen grains to the insect body by allowing a better storage of the pollenkitt. This sticky substance, of which functions are not yet quite understood, is produced by entomogamous species and stored on the surface of the pollen wall [25]. The pollenkitt would enable pollen grains to adhere on the hairs of insects or on the feathers of birds in case of ornithophily [23,24]. In the Araceae however, pollen grains were depicted as poor in pollenkitt [26] and it was suggested that sticky secretions on the stigma and/or the inner spathe surface may play the same role as the pollenkitt [27], accounting for the lack of pollen ornamentation in beetle pollinated species of Araceae.

The fact that no correlations could be detected in the palm family may be due to various reasons. First, it has to be stressed that the sampling was sparser for palms than for the Araceae. In particular, there was a poor overlap at the species level between the pollen and the pollinator datasets. This led us to combine information from different species for the ornamentation type, which we are aware may be questionable considering the high lability of the character even at the intraspecific level. We tried to overcome this problem by attributing polymorphic character states whenever intrageneric diversity was recorded, and by applying two different treatments to polymorphic species in the comparative analyses but we cannot exclude that the choices made here (due to the scarcity of data concerning pollination systems in palms) may have biased the results. However, there is a possibility that our results indeed reflect the reality and that pollen ornamentation is not involved in the pollination syndrome in palms. In this family, the traits linked to pollinator identity still remain almost unknown. Palm flowers are relatively poorly diversified in morphology when compared to the spectacular flowers of other groups [20]. In many species, flowers are visited by many insect families and species (often more than 50 species), although maybe among these insects only a single or a small number of species effectively act as pollinators [28]. The lack of correlation between pollen ornamentation and pollinators may be accounted for by a weak degree of specialization in the pollination system, or it may be that some other factors (like pollenkitt or scents for example) may be dominant in the pollen-pollinator interaction.

In conclusion, it is our feeling that there is little to be gained from seeking a general tendency concerning the relationship between the type of pollen ornamentation and the pollination system across the angiosperms. The ornamentation of the pollen wall is only one of the numerous elements that constitute the pollination syndrome and certainly not the most important [29]. Like the other factors [30], the relative importance of its role in the plant-pollinator interaction may indeed vary among plant taxa or according to geography, and it may have an adaptive value in some groups but evolve in a neutral way in other groups.

## Competing interests

The authors declare that they have no competing interests.

## Authors' contributions

JS performed the coding and the optimization of the reconstruction of the characters, carried out all the comparative analyses, interpreted the data and contributed to writing the manuscript. WJB provided the phylogenetic framework for the Arecaceae. MCA assembled the dataset on the pollination type in Arecaceae. SN supervised the study and contributed to writing the manuscript. All authors read and approved the final manuscript.

## Supplementary Material

Additional file 1 **Studies published about relationship between the ornamentation of pollen grains and others characters in angiosperms**. Studies previously published about relationship between the ornamentation of pollen grains and others characters in angiosperms. Click here for file

Additional file 2 **Detailed information on the methods used**. Detailed information on the material and the methods used in the study. Click here for file

Additional file 3 **Evolution of the ornamentation and of the pollination in Araceae when polymorphic species are duplicated and with the coding 'Psilate/Verrucate' vs. 'Other Ornamentation' and 'Beetle' vs 'Other Pollination'**. A. Optimization of the ornamentation type coded as 'Other-O' (white) and 'Psilate/Verrucate' (black). B. Optimization of the pollination type coded as 'Other-P' (white) and 'Beetle' (black). The bicoloured branches indicate an equivocal inference of the ancestral character state. The transitions towards 'Beetle' pollination and 'Psilate/Verrucate' ornamentation are indicated by full crossbars and the reversals towards 'Other-P' pollination and 'Other-O' ornamentation are indicated by open crossbars (red and blue crossbars correspond respectively to the ACCTRAN and DELTRAN optimizations). Species names are coloured according to the subfamilies (Orontioideae in pink, Monsteroideae in blue, Lasioideae in orange, Calloideae in green and Aroideae in red). Click here for file

Additional file 4 **Evolution of the ornamentation and of the pollination in Araceae when polymorphic species are removed and with the 'Echinulate' vs. 'Other Ornamentation' and 'Fly' vs 'Other Pollination'**. A. Optimization of the ornamentation type coded as 'Other-O' (white) and 'Echinulate' (black). B. Optimization of the pollination type coded as 'Other-P' (white) and 'Fly' (black). The bicoloured branches indicate an equivocal inference of the ancestral character state. The transitions towards 'Fly' pollination and 'Echinulate' ornamentation are indicated by full crossbars and the reversals towards 'Other-P' pollination and 'Other-O' ornamentation are indicated by open crossbars (red and blue crossbars correspond respectively to the ACCTRAN and DELTRAN optimizations). Species names are coloured according to the subfamilies (Orontioideae in pink, Monsteroideae in blue, Lasioideae in orange, Calloideae in green and Aroideae in red). Click here for file

Additional file 5 **Evolution of the ornamentation and of the pollination in Araceae when polymorphic species are duplicated and with the coding 'Echinulate' vs. 'Other Ornamentation' and 'Fly' vs 'Other Pollination'**. A. Optimization of the ornamentation type coded as 'Other-O' (white) and 'Echinulate' (black). B. Optimization of the pollination type coded as 'Other-P' (white) and 'Fly' (black). The bicoloured branches indicate an equivocal inference of the ancestral character state. The transitions towards 'Fly' pollination and 'Echinulate' ornamentation are indicated by full crossbars and the reversals towards 'Other-P' pollination and 'Other-O' ornamentation are indicated by open crossbars (red and blue crossbars correspond respectively to the ACCTRAN and DELTRAN optimizations). Species names are coloured according to the subfamilies (Orontioideae in pink, Monsteroideae in blue, Lasioideae in orange, Calloideae in green and Aroideae in red). Click here for file

Additional file 6 **Detailed results about the comparative analyses conducted with the Concentrated-Changes Test in Araceae. **Pollen ornamentation was coded as 'Psilate/Verrucate' vs. 'Other Ornamentation', pollination system was coded as 'Beetle' vs 'Other Pollination'. A – Distribution of events in the character 'pollination type' on branches reconstructed as having 'Psilate/Verrucate' and 'Other-O' ornamentation, respectively. B – Distribution of events in the character ornamentation type on branches reconstructed as having 'Beetle' and 'Other-P' pollination, respectively. O: Other-P or Other-O depending on the context; B: Beetle; P/V: Psilate/Verrucate; 1: Pollination and ornamentation type reconstructed with ACCTRAN; 2: Pollination and ornamentation type reconstructed with DELTRAN. The Fisher exact test was computed for the columns with numbers in bold (transitions O→B and O→O for table A; O→P/V and O→O for table B). Click here for file

Additional file 7 **Detailed results about the comparative analyses conducted with the Concentrated-Changes Test in Araceae. **Pollen ornamentation was coded as 'Echinulate' vs. 'Other Ornamentation', pollination system was coded as 'Fly' vs 'Other Pollination'. A – Distribution of events in the character 'pollination type' on branches reconstructed as having 'Echinulate' and 'Other-O' ornamentation, respectively. B – Distribution of events in the character ornamentation type on branches reconstructed as having 'Fly' and 'Other-P' pollination, respectively. O: Other-P or Other-O depending on the context; F: Fly; E: Echinulate; 1: Pollination and ornamentation type reconstructed with ACCTRAN; 2: Pollination and ornamentation type reconstructed with DELTRAN. The Fisher exact test was computed for the columns with numbers in bold (transitions O→B and O→O for table A; O→P/V and O→O for table B). Click here for file

Additional file 8 **Evolution of the ornamentation and of the pollination in Arecaceae when polymorphic species are duplicated and with the coding 'Echinulate' vs. 'Other Ornamentation' and 'Fly' vs 'Other Pollination'**. A. Optimization of the ornamentation type coded as 'Other-O' (white) and 'Echinulate' (black). B. Optimization of the pollination type coded as 'Other-P' (white) and 'Fly' (black). The bicoloured branches indicate an equivocal inference of the ancestral character state. The transitions towards 'Fly' pollination and 'Echinulate' ornamentation are indicated by full crossbars and the reversals towards 'Other-P' pollination and 'Other-O' ornamentation are indicated by open crossbars (red and blue crossbars correspond respectively to the ACCTRAN and DELTRAN optimizations). Species names are coloured according to the subfamilies (Calamoideae in blue, Nypoideae in orange, Coryphoideae in green, Ceroxyloideae in pink and Arecoideae in red). Click here for file

Additional file 9 **Detailed results about the comparative analyses conducted with the Concentrated-Changes Test in Arecaceae. **Pollen ornamentation was coded as 'Psilate/Verrucate' vs. 'Other Ornamentation', pollination system was coded as 'Beetle' vs 'Other Pollination'. A – Distribution of events in the character 'pollination type' on branches reconstructed as having 'Psilate/Verrucate' and 'Other-O' ornamentation, respectively. B – Distribution of events in the character ornamentation type on branches reconstructed as having 'Beetle' and 'Other-P' pollination, respectively. O: Other-P or Other-O depending on the context; B: Beetle; P/V: Psilate/Verrucate; 1: Pollination and ornamentation type reconstructed with ACCTRAN; 2: Pollination and ornamentation type reconstructed with DELTRAN. The Fisher exact test was computed for the columns with numbers in bold (transitions O→B and O→O for table A; O→P/V and O→O for table B). Click here for file

Additional file 10 **Detailed results about the comparative analyses conducted with the Concentrated-Changes Test in Arecaceae.** Pollen ornamentation was coded as 'Echinulate' vs. 'Other Ornamentation', pollination system was coded as 'Fly' vs 'Other Pollination'. A – Distribution of events in the character 'pollination type' on branches reconstructed as having 'Echinulate' and 'Other-O' ornamentation, respectively. B – Distribution of events in the character ornamentation type on branches reconstructed as having 'Fly' and 'Other-P' pollination, respectively. O: Other-P or Other-O depending on the context; F: Fly; E: Echinulate; 1: Pollination and ornamentation type reconstructed with ACCTRAN; 2: Pollination and ornamentation type reconstructed with DELTRAN. The Fisher exact test was computed for the columns with numbers in bold (transitions O→B and O→O for table A; O→P/V and O→O for table B). Click here for file
