# Supplementary material for: A comparative analysis of pollinator type and pollen ornamentation in the Araceae and the Arecaceae, two unrelated families of the monocots
Source: BMC Res Notes. 2009 Jul 22;2:145. doi: 10.1186/1756-0500-2-145 (PMC2734846; doi:10.1186/1756-0500-2-145)
Supplement: Additional file 1 — Studies published about relationship between the ornamentation of pollen grains and others characters in angiosperms. Studies previously published about relationship between the ornamentation of pollen grains and others characters in angiosperms. [file 1756-0500-2-145-S1.pdf]

Additional file 1. **Studies published about relationship between the ornamentation of pollen grains and others characters in angiosperms.**

Taxa	Reference	Data type
Angiosperms	Whitehead [1]	Relation between wind-pollination and smooth pollen grains
Polemoniaceae	Taylor and Levin [2]	No apparent association between pollination mechanism and pollen sculpturing
Angiosperms	Heslop-Harrison [3]	Relation between exine and pollenkitt
Angiosperms	Hesse [4]	Relation between exine sculpturing, pollenkitt and pollination syndromes
Papilionoideae	Ferguson and Skvarla [5]	Correlation between pollen morphology and pollination mechanisms
Angiosperms	Zavada [6]	Association between exine sculpturing and self-incompatibility mechanisms
Araceae	Grayum [7]; Grayum [8]	Correlation between exine ornamentation and pollination type
Bauhinia	Ferguson and Pearce [9]	Correlation between exine ornamentation and pollination
Angiosperms	Gibbs and Ferguson [10]	No apparent association between exine sculpturing and self-incompatibility mechanisms
Cabombaceae	Osborn *et al.* [11]	Relation between exine sculpturing, pollenkitt and pollination
Callitrichaceae	Osborn and Philbrick [12]; Osborn *et al.* [13]	Correlation between hydrophily and smooth pollen grains
Angiosperms	Linder [14]	Relation between smooth pollen ornamentation and wind pollination
Angiosperms	Stroo [15]	No association between bat pollinated plants and pollen morphology
Hydrocharitaceae	Tanaka *et al.* [16]	Correlation between pollen morphology and pollination mechanisms
Orchidinae	Lumaga *et al.* [17]	Relation between exine micromorphology and pollination strategies

1. Whitehead DR: **Wind pollination in the angiosperms; evolutionary and environmental considerations**. *Evolution* 1969, **23**:28-35.
2. Taylor TN, Levin DA: **Pollen morphology of Polemoniaceae in relation to systematics and pollination systems: scanning electron microscopy**. *Grana* 1975, **15**:91-112.

3. Heslop-Harrison J: **The adaptative significance of the exine**. In: *The evolutionary significance of the exine.* Edited by Ferguson IK, Muller M. London: Academic press; 1976: 27-37.
4. Hesse M: **The fine structure of the exine in relation to the stickiness of angiosperm pollen**. *Review of Palaeobotany and Palynology* 1981, **35**:81-92.
5. Ferguson IK, Skvarla JJ: **Pollen morphology in relation to pollinators in Papilionoideae (Leguminosae)**. *Botanical Journal of the Linnean Society* 1982, **84**:183-193.
6. Zavada MS: **The relation between pollen exine sculpturing and self-incompatibility mechanisms**. *Plant Systematics and Evolution* 1984, **147**:63-78.
7. Grayum MH: **Correlations between pollination biology and pollen morphology in the Araceae, with some implications for angiosperm evolution**. In: *Pollen and Spores: form and function.* Edited by Blackmore S, Ferguson IK. NY London: Academic Press; 1986: 313-327.
8. Grayum MH: **Comparative external pollen ultrastructure of the Araceae and putatively related taxa**. *Monographs in Systematic Botany of the Missouri Botanical Garden* 1992, **43**:1-167.
9. Ferguson IK, Pearce KJ: **Observation on the pollen morphology of the genus *Bauchinia L.* (Leguminosae: Caesalpinioideae) in the neotropics**. In: *Pollen and spores: form and function.* vol. 12. London, UK: Academic Press, ; 1986: 283-296.
10. Gibbs PE, Ferguson IK: **Correlations between pollen exine sculpturing and angiosperm self-incompatibility systems - a reply**. *Plant Systematics and Evolution* 1987, **157**:143-159.
11. Osborn JM, Taylor TN, Schneider EL: **Pollen morphology and ultrastructure of the Cabombaceae: correlations with pollination biology**. *American Journal of Botany* 1991, **78**(10):1367-1378.
12. Osborn JM, Philbrick CT: **Comparative pollen structure and pollination biology in the *Callitrichaceae***. *Acta botanica Gallica* 1994, **141**(2):257-266.

13. Osborn JM, El-Ghazaly G, Cooper RL: **Development of the exineless pollen wall in *Callitriche truncata* (Callitrichaceae) and the evolution of underwater pollination**. *Plant Systematics and Evolution* 2001, **228**:81-87.
14. Linder HP: **Pollen morphology and wind pollination in Angiosperms**. In: *Pollen and Spores: Morphology and Biology*. Edited by Harley MM, Morton CM, Blackmore S. Kew: Royal Botanic Gardens; 2000: 73-88.
15. Stroo A: **Pollen morphological evolution in bat pollinated plants**. *Plant Systematics and Evolution* 2000, **222**:225-242.
16. Tanaka N, Uehara K, Murata J: **Correlation between pollen morphology and pollination mechanisms in the Hydrocharitaceae**. *Journal of Plant Research* 2004, **117**:265-276.
17. Lumaga MR, Cozzolino S, Kocyan A: **Exine Micromorphology of Orchidinae (Orchidoideae, Orchidaceae): Phylogenetic Constraints or Ecological Influences?** *Annals of Botany* 2006, **98**:237-244.
