# Supplementary material for: A comparative analysis of pollinator type and pollen ornamentation in the Araceae and the Arecaceae, two unrelated families of the monocots
Source: BMC Res Notes. 2009 Jul 22;2:145. doi: 10.1186/1756-0500-2-145 (PMC2734846; doi:10.1186/1756-0500-2-145)
Supplement: Additional file 2 — Detailed information on the methods used. Detailed information on the material and the methods used in the study. [file 1756-0500-2-145-S2.pdf]

Additional file 2: Detailed information on the methods used.

Phylogenetic framework for the Araceae

We used the last published phylogeny of Araceae [1] to infer the relationships among the 33 genera examined in the study of Grayum [2]. All branches were set equal with a value of 1. Polytomies were introduced when phylogenetic relationships between taxa remained unclear. Additional polytomies were included concerning the genera for which relationships among species are still unresolved. Since the software Discrete [3] that we used for the comparative analyses (described hereafter) cannot deal correctly with polytomies, they needed to be removed or resolved. We chose to resolve artificially the polytomies by using the principle of Maximum Parsimony and minimising the number of transitions in each character considered. The branches that resulted from the resolution of polytomies were given a value close to zero (10^{-5}), so that they appeared as negligible in comparison with all other branches.

Phylogenetic framework for the Arecaceae

The topology for the phylogeny of Arecaceae used here was obtained from a Supertree produced recently by Baker *et al.* [4]. This Supertree is based on all previously published data from relevant higher level studies of palms and was built using matrix representation with parsimony [5-7]. It is based on data from 13 DNA regions, a restriction fragment length polymorphism dataset and a morphological dataset. It contains all 192 genera of palms [8] and is almost completely resolved. The genera for which data on the two characters of interest were not available were pruned off the supertree, leaving a total of 43 genera distributed among the five subfamilies recognised in the family (Calamoideae, Nypoideae, Coryphoideae, Ceroxyloideae and Arecoideae). Since the Supertree was constructed via meta-analysis, all branches were attributed a default length of 1. Polytomies due to the lack of phylogenetic information were artificially resolved for the purpose of the comparative analysis as previously described for the Araceae. Here again, a value of 10^{-5} was given to the newly created branches.

Character coding and optimisation

The two characters examined in this study, namely pollen ornamentation (a morphological character) and pollinator type (an ecological character) were coded as discrete variables. Data for the Araceae were obtained from the study of Grayum [2]. For the Arecaceae, data for both characters were extracted from the literature. Concerning pollen ornamentation, we considered only the external aspect of the pollen wall. The internal structure is not taken into account, which is important to stress since its description may depend upon the method used (Light Microscopy, Scanning and Transmission Electron Microscopy on acetolysed pollen or not) contrary to exine sculpturing that remains identical. The table below gives the character states defined for each of the two characters considered. For the type of ornamentation, we used the same coding as Grayum [2]. We grouped the foveolate and

reticulate states into one character state, since he did not relate any of these types to a particular type of pollinator.
In the Arecaceae, there was almost no overlap at the species level between the dataset on pollen ornamentation and the dataset on pollinator type. Our strategy was to keep all genera for which information was available from the literature for both characters. However, pollen ornamentation is a rather labile character and very often, various pollen ornamentations are described for different species within the same genus. In consequence, for the species of our dataset which were not described for pollen ornamentation, we chose to take into account all the character states described within the genus to which they belong.

Concerning the pollinator type, we used the four character states described by Grayum [2] in the Araceae (‘Beetles’, ‘Flies’, ‘Bees’ and ‘Thrips’). Two additional character states were needed for the Arecaceae, namely ‘Wind’ and ‘Bats’. Intraspecific polymorphism was reported for this character in several species.

Character optimization on the trees was carried out with the Maximum Parsimony method (MP) implemented in the software Mesquite [9]. The character states were unordered and polytomies were interpreted as “soft” (uncertainty in resolution). The Maximum Likelihood method (ML) is also available in this software but was not employed here for two reasons: (1) this method is optimal when branch lengths are known, which is not the case here: (2) this method does not accept polymorphisms, which were numerous in our dataset.

Since multistate characters could not be used for the comparative analyses, we transformed them into binary characters. In order to make up for the loss of information due to the combination of the different character states into two states only, we produced two different binary codings to conduct the comparative analyses. Ornamentation type was coded as ‘Psilate/Verrucate’ (state 1) versus ‘Other ornamentation’ (state 0) as a first option and as ‘Echinulate’ (state 1) versus ‘Other ornamentation’ (state 0) as a second option. Pollinator type was coded as ‘Beetles/Wind’ (state 1) versus ‘Other pollination’ (state 0) as a first option and as ‘Flies’ (state 1) versus ‘Other pollination’ (state 0) as a second option. These two binary codings enabled us to test the two correlations suggested by Grayum [2]: i) between psilate/verrucate ornamentation and beetle pollination and ii) between echinulate ornamentation and fly pollination. We chose to combine beetles and wind in the first option since it has been demonstrated in several groups that wind pollination is closely related to psilate ornamentation [10, 11]. The softwares that were used in this study for the comparative analyses do not accept polymorphism in terminal taxa. To eliminate these polymorphisms we tried two different strategies: polymorphic species were either removed, or duplicated. In the latter situation, polymorphic

branches were split into two branches that were each given a length of 10^{-5}. Species names are followed by numbers 1 and 2 in such cases.

Phylogenetic signal

According to some authors, Phylogenetic Comparative Methods should be used only when the data are partly dependent upon the phylogeny [12, 13]. This can be achieved through the detection of a phylogenetic signal in each of the character studied. This method consists of testing whether the data are randomly distributed on the phylogeny [14]. If not, a phylogenetic signal is detected and a Phylogenetic Comparative Method (PCM) can be performed. This technique has been successfully employed several times in a context of character optimization [14-19]. If character states are randomly distributed among taxa, the maximum parsimony method does not give a reliable evaluation of ancestral character states. The distribution of the ancestral character states can also be random. Testing the phylogenetic signal has thus two utilities: (i) to measure the reliability of character optimization and (ii) to indicate the relevance of using a PCM. The test consists of simulating random trees and counting for each of them the number of transitions required to account for the observed distribution of character states. If the number of transition in 95% of the randomly generated trees is higher than the number of transition observed on the actual phylogeny, we conclude that there is a phylogenetic signal. 10 000 simulations of random trees were carried out using the TreeFarm package of Mesquite [9] providing a distribution of the number of steps required to explain the distribution of character states under the hypothesis of no phylogenetic signal. The uniform model of speciation (model of Yule) was used with a tree depth fixed by default at 10. Character states always remained associated to their taxon.

Phylogenetic Comparative Analyses

Two methods of comparative analysis of discrete characters were used in this study: the Concentrated Changes Test or CCT [20] and Discrete [3]. The first method is based on Maximum Parsimony and relies on ancestral states inference, whereas the second method is based on Maximum Likelihood and does require any inference of ancestral states.

The CCT tests whether changes in the character considered as dependent are associated phylogenetically and significantly with a particular state of the independent character. This test requires that all branches of the tree have unequivocal character states but allows for polytomies. In order to solve the equivocal ancestral states inferred by the MP method, two different types of optimization were carried out for each of the two characters. Optimization 1 maximizes the number of reversals (ACCTRAN) whereas the Optimization 2 maximizes the number of convergences (DELTRAN). The transitions ($0 \rightarrow 1$) and ($0 \rightarrow 0$) for the dependent character are counted in the branches where the independent character has state 0 and in the branches where it has state 1. An exact

Fischer's test (2 x 2 table) is carried out under the null hypothesis that whatever the state of the independent character, state 1 of the dependent character is equally likely to evolve [21].

The discrete variable method of Pagel [3] is implemented in the software Discrete available from www.evolution.rdg.ac.uk [3, 22] and uses a maximum likelihood model to characterize evolutionary changes along each branch of a phylogenetic tree. This method requires a fully resolved phylogenetic tree. A likelihood ratio is computed to test for correlated evolution between two binary characters. The likelihoods compared through this ratio are obtained from two different models of evolution applied to data. One of these models describes an independent evolution of the two traits (H_0) while the other describes a correlated evolution between these two traits (H_1) (Omnibus test). If the dependent model fits better the data than the independent one, then the two characters can be considered as having evolved in a correlated way. The likelihood ratio test enables comparison between the goodness of fit of the two models, with LR = $-2\log_e$ [L(H0)/L(H1)]. The likelihood ratio is asymptotically chi-square distributed with df = 4, where degrees of freedom equal the difference in the number of parameters between the two models. There are four parameters in the independent model versus eight in the dependent one [3]. The LR is then compared with χ^2 ($\chi^2_4 = 9.49$ with a confidence threshold of 5%). Discrete also permits the testing of the temporal ordering and direction of evolutionary changes (*i.e.*, contingent change test) of the two variables on the phylogeny. In these tests, one of the eight parameters of the model is constrained and the reduced model of seven parameters is compared to the full model of eight parameters. The LR is asymptotically chi-square distributed with df=1 and is compared with χ^2 (χ^2_1=3.84 with a confidence threshold of 5%). It is also possible, when appropriate, to test if the various changes are significantly different from zero (temporal order test). The results of the contingent change test and the temporal order test can be summarized in a flow diagram.

In our study, each character (ornamentation type and pollination type) was considered as the independent trait in turn, in order to compare both situations without any particular assumption *a priori*.

List of characters and character states examined in this study.

Character	State 0	State 1	State 2	State 3	State 4	State 5
Pollen ornamentation	Psilate	Verrucate	Striate	Echinulate	Foveolate/Reticulate	Finely perforate/Rugulate
Pollination type	Beetle	Fly	Bee	Thrip	Bat	Wind

Pollen ornamentation is coded according to the distinctions made by Grayum [2]. The Echinulate state corresponds to the bacculate and gemnate pollen grains in Arecaceae. Foveolate and reticulate pollen grains are considered as presenting similar characteristics for this study.

1. Cabrera LI, Salazar GA, Chase MW, Mayo SJ, Bogner J, Davila P: **Phylogenetic relationships of Aroids and Duckweeds (Araceae) inferred from coding and noncoding plastid DNA**. *American Journal of Botany* 2008, **95**(9):1153-1165.
2. Grayum MH: **Correlations between pollination biology and pollen morphology in the Araceae, with some implications for angiosperm evolution**. In: *Pollen and Spores: form and function.* Edited by Blackmore S, Ferguson IK. NY London: Academic Press; 1986: 313-327.
3. Pagel M: **Detecting correlated evolution on phylogenies: a general method for the comparative analysis of discrete characters**. *Proceedings of the Royal Society of London Series B: Biological Sciences* 1994, **255**(37-45).
4. Baker WJ, Wilkinson M, Asmussen-Lange CB, Chase MW, Dransfield J, Forest F, Harley MM, Uhl NW, Savolainen V: **A complete generic level phylogeny of palms (Arecaceae) with comparisons of supertree and supermatrix approaches**. *Syst Biol* In Review.
5. Baum BR: **Combining trees as a way of combining datasets for phylogenetic inferences, and the desirability of combining gene trees**. *Taxon* 1992, **41**:3-10.
6. Baum BR, Ragan MA: **A comment on Baum's method for combining phylogenetic trees-reply**. *Taxon* 1993, **42**:637-640.
7. Ragan MA: **Phylogenetic inference based on matrix representation of trees**. *Molecular Phylogenetics and Evolution* 1992, **1**:53-58.
8. Govaerts R, Dransfield J: **World checklist of palms**. Kew: Royal Botanic Gardens; 2005.
9. Maddison WP, Maddison DR: **Mesquite: a modular system for evolutionary analysis**. In., Version 2.0 edn; 2006.
10. Walker JW: **Evolution of exine structure in the pollen of primitive angiosperms**. *American Journal of Botany* 1974, **61**:891-902.
11. Osborn JM, Taylor TN, Schneider EL: **Pollen morphology and ultrastructure of the Cabombaceae: correlations with pollination biology**. *American Journal of Botany* 1991, **78**(10):1367-1378.
12. Björklund M: **Are 'comparative methods' always necessary?** *Oikos* 1997, **80**(3):607-612.
13. Abouheif E: **A method for testing the assumption of phylogenetic independence in comparative data**. *Evolutionary Ecology Research* 1999, **1**:895-909.

14. Laurin M: **The evolution of body size, Cope's rule and the origin of Amniotes**. *Systematic Biology* 2004, **53**(4):594-622.
15. Cubo J, Ponton F, Laurin M, De Margerie E, Castanet J: **Phylogenetic signal in bone microstructure of Sauropsids**. *Systematic Biology* 2005, **54**(4):562-574.
16. Germain D, Laurin M: **Microanatomy of the radius and lifestyle in amniotes (Vertebrata, Tetrapoda)**. *Zoologica Scripta* 2005, **34**(4):335-350.
17. Laurin M, Girondot M, Loth M-M: **The evolution of long bone microstructure and lifestyle in lissamphibians**. *Paleobiology* 2004, **30**(4):589-613.
18. Penet L, Nadot S, Ressayre A, Forchioni A, Dreyer L, Gouyon PH: **Multiple developmental pathways leading to a single morph: monosulcate pollen (examples from the Asparagales)**. *Annals of Botany* 2005, **95**:331-343.
19. Sannier J, Nadot S, Forchioni A, Harley MM, Albert B: **Variations in the microsporogenesis of monosulcate palm pollen**. *Botanical Journal of the Linnean Society* 2006, **151**(1):93-102.
20. Maddison WP: **A method for testing the correlated evolution of txo binary characters: are gains or losses concentrated on certain branches of a phylogenetic tree?** *Evolution* 1990, **44**(3):539-557.
21. Sillén-Tullberg B: **The effect of biaised inclusion of taxa on the correlation between discrete characters in phylogenetic trees**. *Evolution* 1993, **47**(4):1182-1191.
22. Pagel M: **Inferring the historical patterns of biological evolution**. *Nature* 1999, **401**:877-884.
