# Supplementary material for: A comparative analysis of pollinator type and pollen ornamentation in the Araceae and the Arecaceae, two unrelated families of the monocots
Source: BMC Res Notes. 2009 Jul 22;2:145. doi: 10.1186/1756-0500-2-145 (PMC2734846; doi:10.1186/1756-0500-2-145)
Supplement: Additional file 3 — Evolution of the ornamentation and of the pollination in Araceae when polymorphic species are duplicated and with the coding 'Psilate/Verrucate' vs. 'Other Ornamentation' and 'Beetle' vs 'Other Pollination'. A. Optimization of the ornamentation type coded as 'Other-O' (white) and 'Psilate/Verrucate' (black). B. Optimization of the pollination type coded as 'Other-P' (white) and 'Beetle' (black). The bicoloured branches indicate an equivocal inference of the ancestral character state. The transitions towards 'Beetle' pollination and 'Psilate/Verrucate' ornamentation are indicated by full crossbars and the reversals towards 'Other-P' pollination and 'Other-O' ornamentation are indicated by open crossbars (red and blue crossbars correspond respectively to the ACCTRAN and DELTRAN optimizations). Species names are coloured according to the subfamilies (Orontioideae in pink, Monsteroideae in blue, Lasioideae in orange, Calloideae in green and Aroideae in red). [file 1756-0500-2-145-S3.pdf]

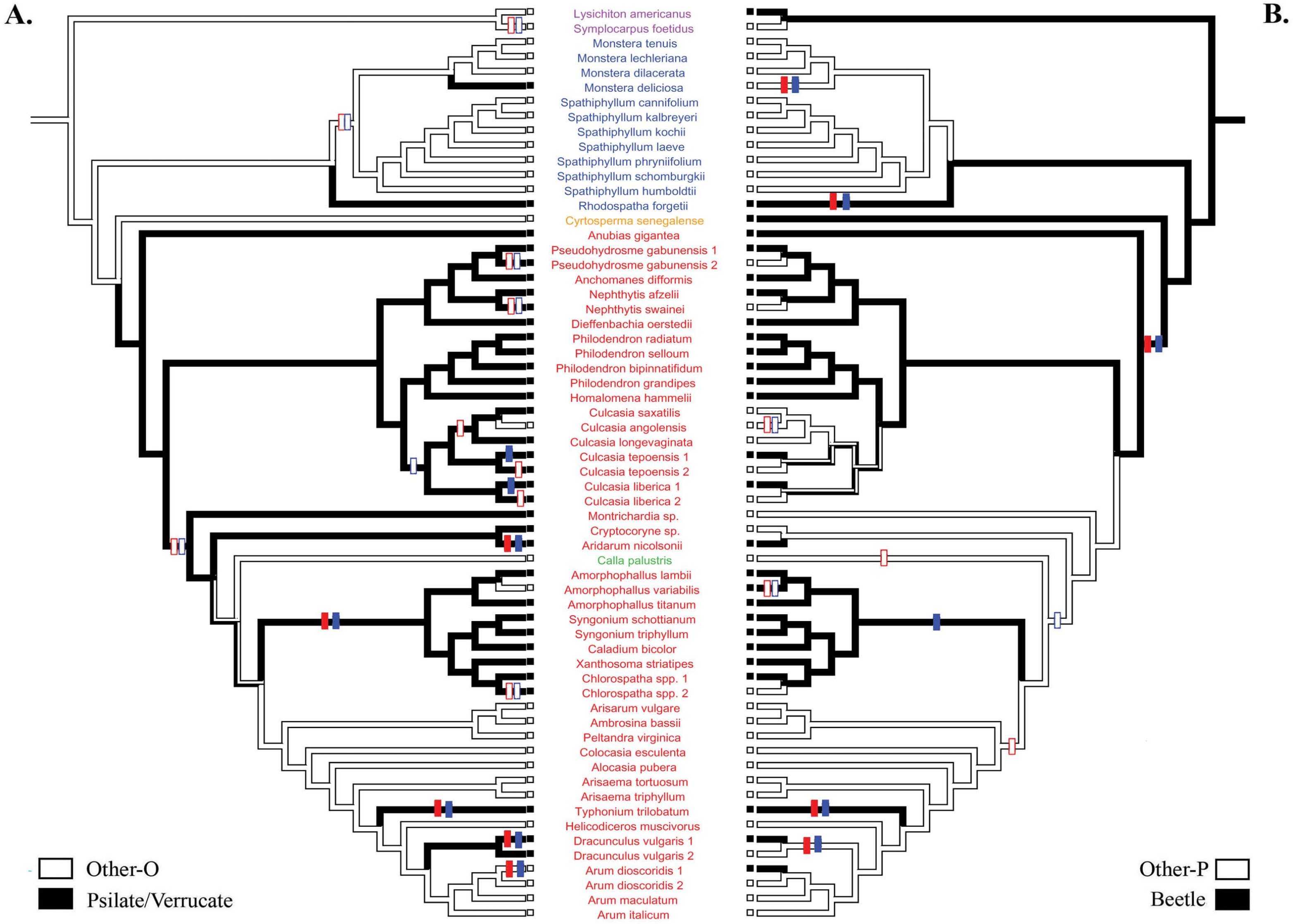
A.
B.
Lysichiton americanus
Symplocarpus foetidus
Monstera tenuis
Monstera lechleriana
Monstera dilacerata
Monstera deliciosa
Spathiphyllum cannifolium
Spathiphyllum kalbreyeri
Spathiphyllum kochii
Spathiphyllum laeve
Spathiphyllum phryniifolium
Spathiphyllum schomburgkii
Spathiphyllum humboldtii
Rhodospatha forgetii
Cyrtosperma senegalense
Anubias gigantea
Pseudohydrosme gabunensis 1
Pseudohydrosme gabunensis 2
Anchomanes difformis
Nephthytis afzelii
Nephthytis swainei
Dieffenbachia oerstedii
Philodendron radiatum
Philodendron selloum
Philodendron bipinnatifidum
Philodendron grandipes
Homalomena hammelii
Culcasia saxatilis
Culcasia angolensis
Culcasia longevaginata
Culcasia tepoensis 1
Culcasia tepoensis 2
Culcasia liberica 1
Culcasia liberica 2
Montrichardia sp.
Cryptocoryne sp.
Aridarum nicolsonii
Calla palustris
Amorphophallus lambii
Amorphophallus variabilis
Amorphophallus titanum
Syngonium schottianum
Syngonium triphyllum
Caladium bicolor
Xanthosoma striatipes
Chlorospatha spp. 1
Chlorospatha spp. 2
Arisarum vulgare
Ambrosina bassii
Peltandra virginica
Colocasia esculenta
Alocasia pubera
Arisaema tortuosum
Arisaema triphyllum
Typhonium trilobatum
Helicodiceros muscivorus
Dracunculus vulgaris 1
Dracunculus vulgaris 2
Arum dioscoridis 1
Arum dioscoridis 2
Arum maculatum
Arum italicum
Other-O
Psilate/Verrucate
Other-P
Beetle
