# Supplementary figures and images for: A comparative analysis of pollinator type and pollen ornamentation in the Araceae and the Arecaceae, two unrelated families of the monocots
Source: BMC Res Notes. 2009 Jul 22;2:145. doi: 10.1186/1756-0500-2-145 (PMC2734846; doi:10.1186/1756-0500-2-145)

A.

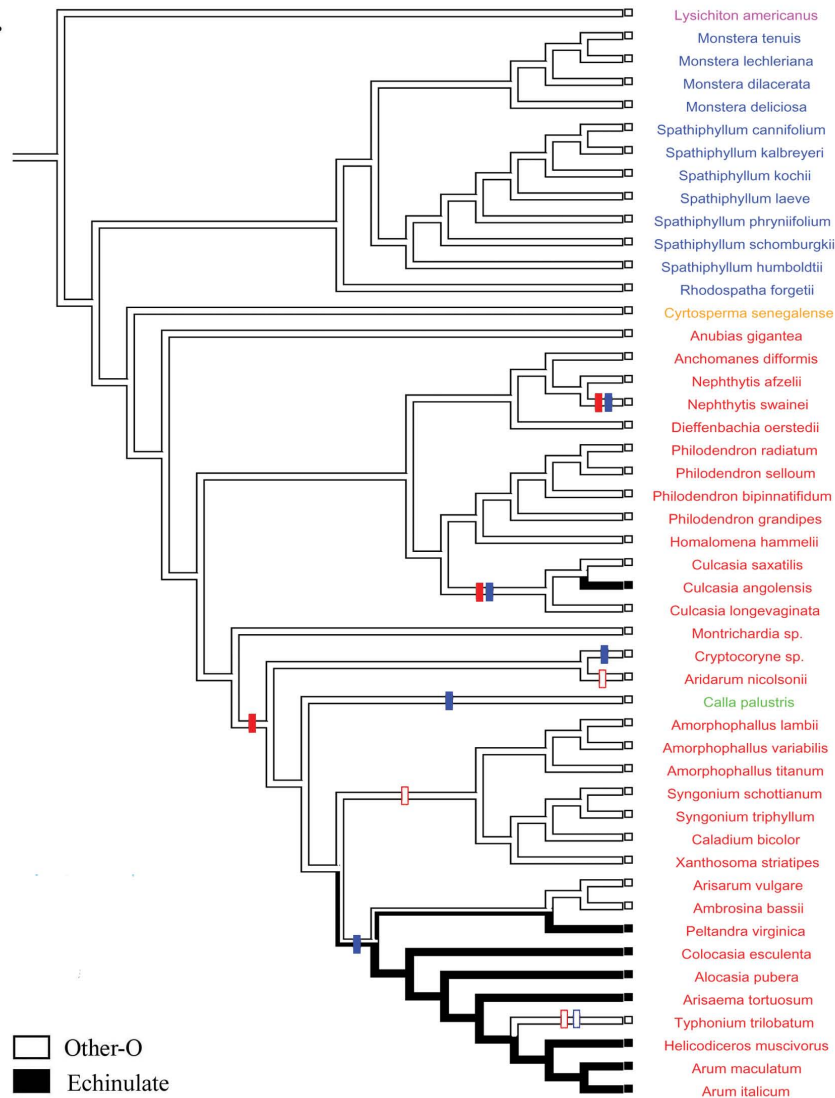

B.

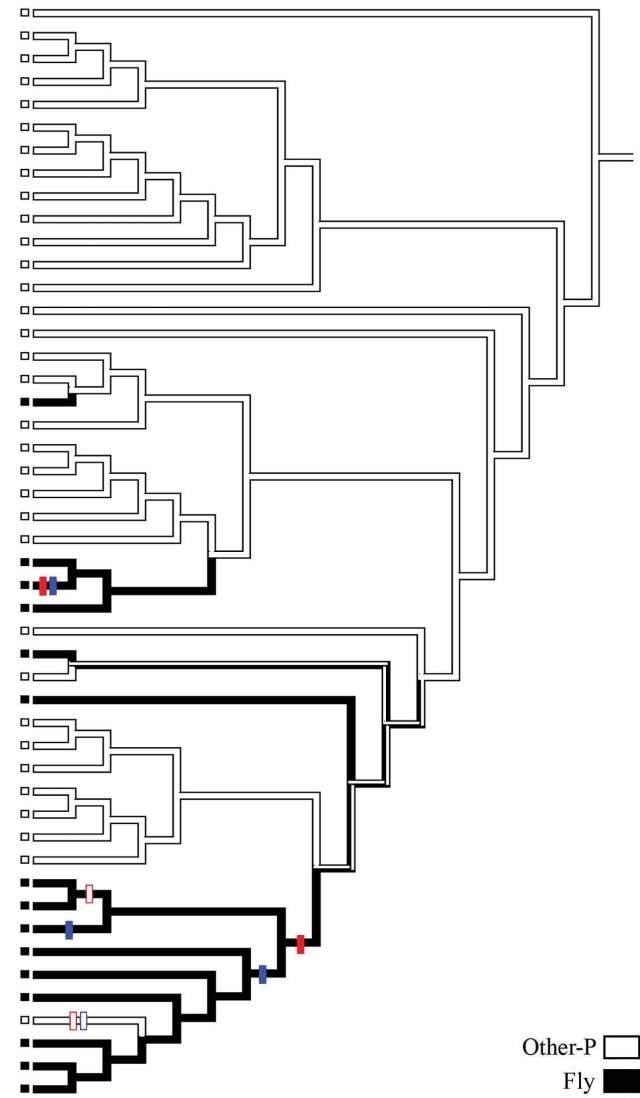

Supplement: Additional file 4 — Evolution of the ornamentation and of the pollination in Araceae when polymorphic species are removed and with the 'Echinulate' vs. 'Other Ornamentation' and 'Fly' vs 'Other Pollination'. A. Optimization of the ornamentation type coded as 'Other-O' (white) and 'Echinulate' (black). B. Optimization of the pollination type coded as 'Other-P' (white) and 'Fly' (black). The bicoloured branches indicate an equivocal inference of the ancestral character state. The transitions towards 'Fly' pollination and 'Echinulate' ornamentation are indicated by full crossbars and the reversals towards 'Other-P' pollination and 'Other-O' ornamentation are indicated by open crossbars (red and blue crossbars correspond respectively to the ACCTRAN and DELTRAN optimizations). Species names are coloured according to the subfamilies (Orontioideae in pink, Monsteroideae in blue, Lasioideae in orange, Calloideae in green and Aroideae in red). [file 1756-0500-2-145-S4.pdf]

A.

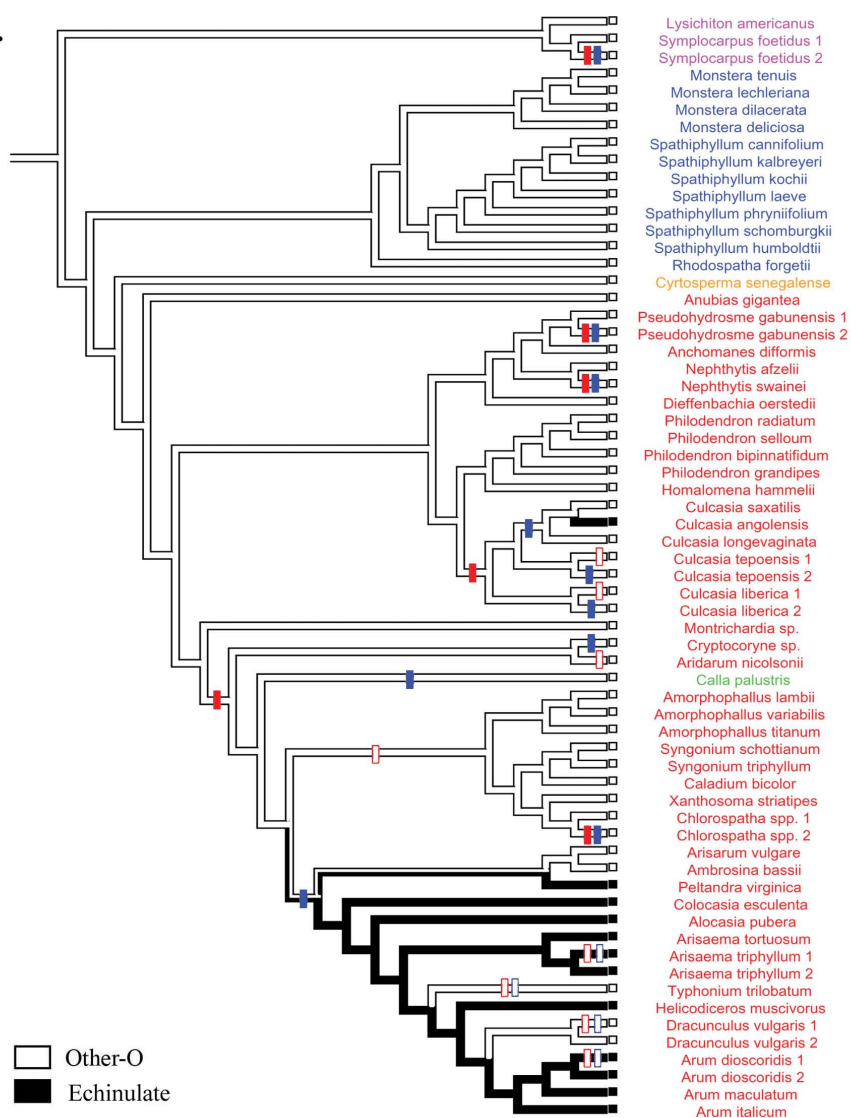

B.

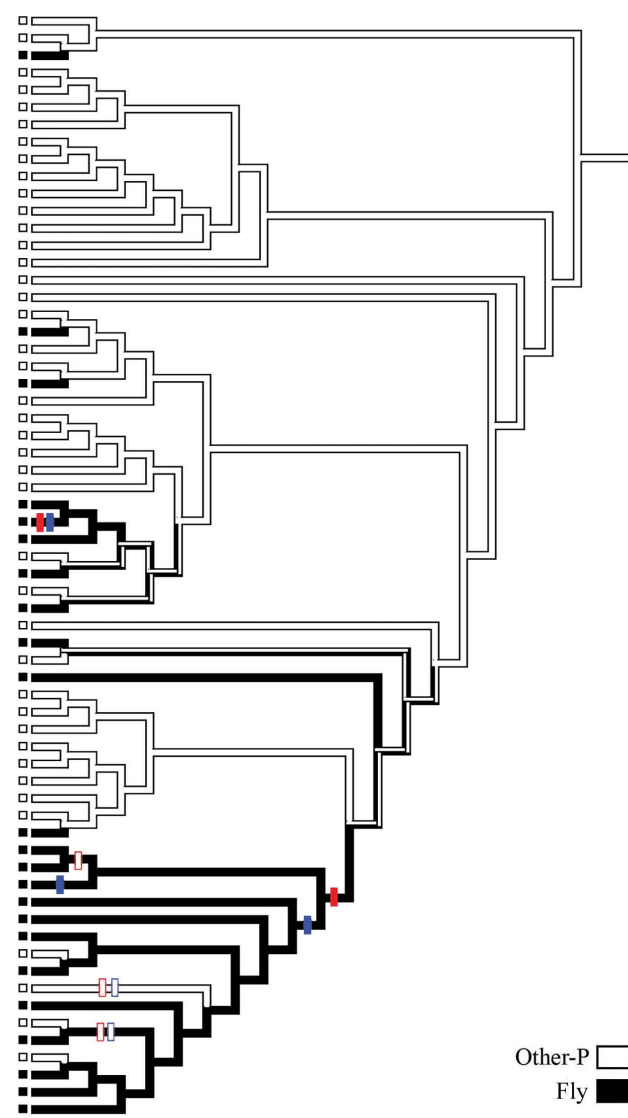

Supplement: Additional file 5 — Evolution of the ornamentation and of the pollination in Araceae when polymorphic species are duplicated and with the coding 'Echinulate' vs. 'Other Ornamentation' and 'Fly' vs 'Other Pollination'. A. Optimization of the ornamentation type coded as 'Other-O' (white) and 'Echinulate' (black). B. Optimization of the pollination type coded as 'Other-P' (white) and 'Fly' (black). The bicoloured branches indicate an equivocal inference of the ancestral character state. The transitions towards 'Fly' pollination and 'Echinulate' ornamentation are indicated by full crossbars and the reversals towards 'Other-P' pollination and 'Other-O' ornamentation are indicated by open crossbars (red and blue crossbars correspond respectively to the ACCTRAN and DELTRAN optimizations). Species names are coloured according to the subfamilies (Orontioideae in pink, Monsteroideae in blue, Lasioideae in orange, Calloideae in green and Aroideae in red). [file 1756-0500-2-145-S5.pdf]

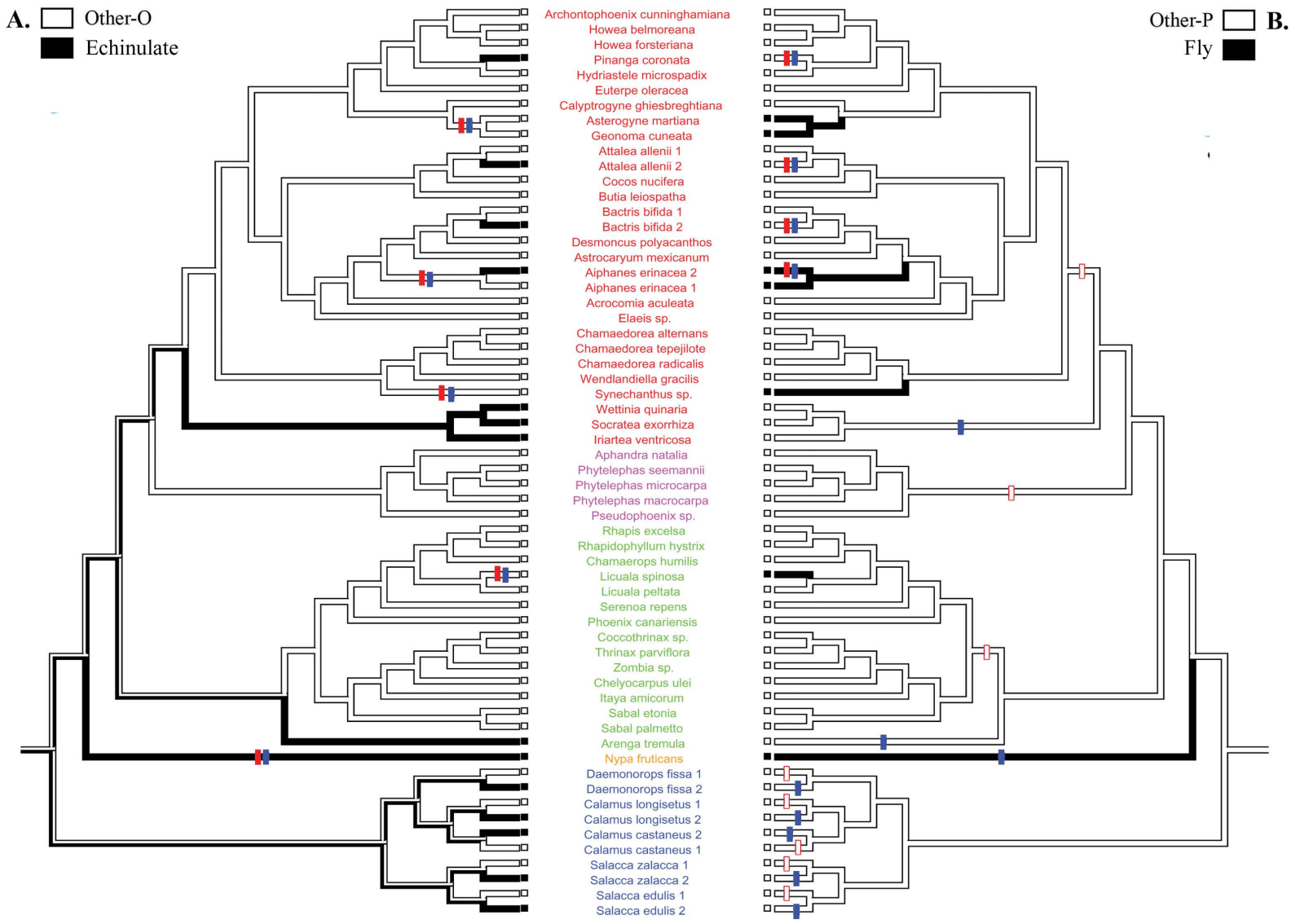

Supplement: Additional file 8 — Evolution of the ornamentation and of the pollination in Arecaceae when polymorphic species are duplicated and with the coding 'Echinulate' vs. 'Other Ornamentation' and 'Fly' vs 'Other Pollination'. A. Optimization of the ornamentation type coded as 'Other-O' (white) and 'Echinulate' (black). B. Optimization of the pollination type coded as 'Other-P' (white) and 'Fly' (black). The bicoloured branches indicate an equivocal inference of the ancestral character state. The transitions towards 'Fly' pollination and 'Echinulate' ornamentation are indicated by full crossbars and the reversals towards 'Other-P' pollination and 'Other-O' ornamentation are indicated by open crossbars (red and blue crossbars correspond respectively to the ACCTRAN and DELTRAN optimizations). Species names are coloured according to the subfamilies (Calamoideae in blue, Nypoideae in orange, Coryphoideae in green, Ceroxyloideae in pink and Arecoideae in red). [file 1756-0500-2-145-S8.pdf]
