# Supplementary material for: A comparative analysis of pollinator type and pollen ornamentation in the Araceae and the Arecaceae, two unrelated families of the monocots
Source: BMC Res Notes. 2009 Jul 22;2:145. doi: 10.1186/1756-0500-2-145 (PMC2734846; doi:10.1186/1756-0500-2-145)
Supplement: Additional file 7 — Detailed results about the comparative analyses conducted with the Concentrated-Changes Test in Araceae. Pollen ornamentation was coded as 'Echinulate' vs. 'Other Ornamentation', pollination system was coded as 'Fly' vs 'Other Pollination'. A – Distribution of events in the character 'pollination type' on branches reconstructed as having 'Echinulate' and 'Other-O' ornamentation, respectively. B – Distribution of events in the character ornamentation type on branches reconstructed as having 'Fly' and 'Other-P' pollination, respectively. O: Other-P or Other-O depending on the context; F: Fly; E: Echinulate; 1: Pollination and ornamentation type reconstructed with ACCTRAN; 2: Pollination and ornamentation type reconstructed with DELTRAN. The Fisher exact test was computed for the columns with numbers in bold (transitions O→B and O→O for table A; O→P/V and O→O for table B). [file 1756-0500-2-145-S7.pdf]

Additional file 7. Comparative analyses conducted with the Concentrated-Changes Test [1] in Araceae. Pollen ornamentation was coded as 'Echinulate' vs. 'Other Ornamentation', pollination system was coded as 'Fly' vs 'Other Pollination'.

A.

			O→B	O→O	B→O	B→B	Fisher Exact Test
			ACCTRAN optimization				
Polymorphic species removed	1	Psilate/Verrucate	**0**	**0**	0	16	NS
		Other ornamentation	**3**	**61**	3	11	
	2	Psilate/Verrucate	**0**	**0**	0	14	NS
		Other ornamentation	**3**	**61**	3	13	
			DELTRAN optimization				
	1	Psilate/Verrucate	**1**	**0**	0	15	NS
		Other ornamentation	**4**	**67**	1	6	
	2	Psilate/Verrucate	**0**	**0**	0	14	NS
		Other ornamentation	**5**	**67**	1	7	
			ACCTRAN optimization				
Polymorphic species duplicated	1	Psilate/Verrucate	**0**	**0**	2	23	NS
		Other ornamentation	**6**	**70**	6	19	
	2	Psilate/Verrucate	**0**	**0**	2	21	NS
		Other ornamentation	**6**	**70**	6	21	
			DELTRAN optimization				
	1	Psilate/Verrucate	**1**	**0**	2	22	NS
		Other ornamentation	**9**	**82**	2	8	
	2	Psilate/Verrucate	**0**	**0**	2	21	NS
		Other ornamentation	**10**	**82**	2	9	

B.

			O→P/V	O→O	P/V→O	P/V→P/V	Fisher Exact Test
			ACCTRAN optimization				
Polymorphic species removed	1	Beetle	**2**	**13**	1	14	P < 0.05
		Other pollination	**0**	**63**	1	0	
	2	Beetle	**2**	**9**	1	14	P < 0.05
		Other pollination	**0**	**67**	1	0	
			DELTRAN optimization				
	1	Beetle	**3**	**16**	0	11	P < 0.05
		Other pollination	**0**	**63**	1	0	

	2	Beetle	**3**	**12**	0	11	P < 0.01
		Other pollination	**0**	**67**	1	0	
				ACCTRAN optimization			
Polymorphic species duplicated	1	Beetle	**2**	**23**	2	21	NS
		Other pollination	**0**	**75**	1	2	
	2	Beetle	**2**	**15**	2	21	P < 0.05
		Other pollination	**0**	**83**	1	2	
				DELTRAN optimization			
	1	Beetle	**3**	**26**	1	18	P < 0.05
		Other pollination	**0**	**75**	1	2	
	2	Beetle	**3**	**18**	1	18	P < 0.01
		Other pollination	**0**	**83**	1	2	

A - Distribution of events in the character ‘pollination type’ on branches reconstructed as having ‘Echinulate’ and ‘Other-O’ ornamentation, respectively. B - Distribution of events in the character ornamentation type on branches reconstructed as having ‘Fly’ and ‘Other-P’ pollination, respectively. O: Other-P or Other-O depending on the context; F: Fly; E: Echinulate; 1: Pollination and ornamentation type reconstructed with ACCTRAN; 2: Pollination and ornamentation type reconstructed with DELTRAN. The Fisher exact test was computed for the columns with numbers in bold (transitions O→B and O→O for table A; O→P/V and O→O for table B).

1. Maddison WP: **A method for testing the correlated evolution of txo binary characters: are gains or losses cencentrated on certain branches of a phylogenetic tree?** *Evolution* 1990, **44**(3):539-557.
