# Supplementary material for: A comparative analysis of pollinator type and pollen ornamentation in the Araceae and the Arecaceae, two unrelated families of the monocots
Source: BMC Res Notes. 2009 Jul 22;2:145. doi: 10.1186/1756-0500-2-145 (PMC2734846; doi:10.1186/1756-0500-2-145)
Supplement: Additional file 9 — Detailed results about the comparative analyses conducted with the Concentrated-Changes Test in Arecaceae. Pollen ornamentation was coded as 'Psilate/Verrucate' vs. 'Other Ornamentation', pollination system was coded as 'Beetle' vs 'Other Pollination'. A – Distribution of events in the character 'pollination type' on branches reconstructed as having 'Psilate/Verrucate' and 'Other-O' ornamentation, respectively. B – Distribution of events in the character ornamentation type on branches reconstructed as having 'Beetle' and 'Other-P' pollination, respectively. O: Other-P or Other-O depending on the context; B: Beetle; P/V: Psilate/Verrucate; 1: Pollination and ornamentation type reconstructed with ACCTRAN; 2: Pollination and ornamentation type reconstructed with DELTRAN. The Fisher exact test was computed for the columns with numbers in bold (transitions O→B and O→O for table A; O→P/V and O→O for table B). [file 1756-0500-2-145-S9.pdf]

Additional file 9. Comparative analyses conducted with the Concentrated-Changes Test [1] in Arecaceae. Pollen ornamentation was coded as ‘Psilate/Verrucate’ vs. ‘Other Ornamentation’, pollination system was coded as ‘Beetle’ vs ‘Other Pollination’.

A.

			O→B	O→O	B→O	B→B	Fisher Exact Test
			ACCTRAN optimization				
Polymorphic species duplicated	1	Psilate/Verrucate	**0**	**5**	1	5	NS
		Other ornamentation	**8**	**41**	5	55	
	2	Psilate/Verrucate	**0**	**5**	1	5	NS
		Other ornamentation	**8**	**41**	5	55	
			DELTRAN optimization				
	1	Psilate/Verrucate	**0**	**5**	1	5	NS
		Other ornamentation	**12**	**51**	1	45	
	2	Psilate/Verrucate	**0**	**5**	1	5	NS
		Other ornamentation	**12**	**51**	1	45	

B.

			O→P/V	O→O	P/V→O	P/V→P/V	Fisher Exact Test
			ACCTRAN optimization				
Polymorphic species duplicated	1	Beetle	**5**	**63**	0	0	NS
		Other pollination	**6**	**46**	0	0	
	2	Beetle	**5**	**57**	0	0	NS
		Other pollination	**6**	**52**	0	0	
			DELTRAN optimization				
	1	Beetle	**5**	**63**	0	0	NS
		Other pollination	**6**	**46**	0	0	
	2	Beetle	**5**	**57**	0	0	NS
		Other pollination	**6**	**52**	0	0	

A - Distribution of events in the character ‘pollination type’ on branches reconstructed as having ‘Psilate/Verrucate’ and ‘Other-O’ ornamentation, respectively. B - Distribution of events in the character ornamentation type on branches reconstructed as having ‘Beetle’ and ‘Other-P’ pollination, respectively. O: Other-P or Other-O depending on the context; B: Beetle; P/V: Psilate/Verrucate; 1: Pollination and ornamentation type reconstructed with ACCTRAN; 2: Pollination and ornamentation type reconstructed with DELTRAN. The Fisher exact test was computed for the columns with numbers in bold (transitions O→B and O→O for table A; O→P/V and O→O for table B).

1. Maddison WP: **A method for testing the correlated evolution of txo binary characters: are gains or losses cencentrated on certain branches of a phylogenetic tree?** *Evolution* 1990, **44**(3):539-557.
