# Supplementary material for: A comparative analysis of pollinator type and pollen ornamentation in the Araceae and the Arecaceae, two unrelated families of the monocots
Source: BMC Res Notes. 2009 Jul 22;2:145. doi: 10.1186/1756-0500-2-145 (PMC2734846; doi:10.1186/1756-0500-2-145)
Supplement: Additional file 10 — Detailed results about the comparative analyses conducted with the Concentrated-Changes Test in Arecaceae. Pollen ornamentation was coded as 'Echinulate' vs. 'Other Ornamentation', pollination system was coded as 'Fly' vs 'Other Pollination'. A – Distribution of events in the character 'pollination type' on branches reconstructed as having 'Echinulate' and 'Other-O' ornamentation, respectively. B – Distribution of events in the character ornamentation type on branches reconstructed as having 'Fly' and 'Other-P' pollination, respectively. O: Other-P or Other-O depending on the context; F: Fly; E: Echinulate; 1: Pollination and ornamentation type reconstructed with ACCTRAN; 2: Pollination and ornamentation type reconstructed with DELTRAN. The Fisher exact test was computed for the columns with numbers in bold (transitions O→B and O→O for table A; O→P/V and O→O for table B). [file 1756-0500-2-145-S10.pdf]

Additional file 10. Comparative analyses conducted with the Concentrated-Changes Test [1] in Arecaceae. Pollen ornamentation was coded as 'Echinulate' vs. 'Other Ornamentation', pollination system was coded as 'Fly' vs 'Other Pollination'.

A.

			O→B	O→O	B→O	B→B	Fisher Exact Test
			ACCTRAN optimization				
Polymorphic species duplicated	1	Psilate/Verrucate	**1**	**28**	0	1	NS
		Other ornamentation	**4**	**81**	0	3	
	2	Psilate/Verrucate	**1**	**14**	0	1	NS
		Other ornamentation	**4**	**95**	0	3	
			DELTRAN optimization				
	1	Psilate/Verrucate	**1**	**28**	0	1	NS
		Other ornamentation	**4**	**81**	0	3	
	2	Psilate/Verrucate	**1**	**14**	0	1	NS
		Other ornamentation	**4**	**95**	0	3	

B.

			O→P/V	O→O	P/V→O	P/V→P/V	Fisher Exact Test
			ACCTRAN optimization				
Polymorphic species duplicated	1	Beetle	**1**	**7**	0	1	NS
		Other pollination	**3**	**73**	8	25	
	2	Beetle	**1**	**7**	0	1	NS
		Other pollination	**3**	**73**	8	25	
			DELTRAN optimization				
	1	Beetle	**2**	**7**	0	0	NS
		Other pollination	**10**	**95**	0	4	
	2	Beetle	**2**	**7**	0	0	NS
		Other pollination	**10**	**95**	0	4	

A - Distribution of events in the character 'pollination type' on branches reconstructed as having 'Echinulate' and 'Other-O' ornamentation, respectively. B - Distribution of events in the character ornamentation type on branches reconstructed as having 'Fly' and 'Other-P' pollination, respectively. O: Other-P or Other-O depending on the context; F: Fly; E: Echinulate; 1: Pollination and ornamentation type reconstructed with ACCTRAN; 2: Pollination and ornamentation type reconstructed with DELTRAN. The Fisher exact test was computed for the columns with numbers in bold (transitions O→B and O→O for table A; O→P/V and O→O for table B).

1. Maddison WP: **A method for testing the correlated evolution of txo binary characters: are gains or losses cencentrated on certain branches of a phylogenetic tree?** *Evolution* 1990, **44**(3):539-557.
